# Correction: Medical Emergency During Flight: A Team-Building Exercise

**DOI:** 10.15766/mep_2374-8265.11186

**Published:** 2021-08-02

## Correction

In the publication “Medical Emergency During Flight: A Team-Building Exercise,” the authors' degrees were stated incorrectly in the byline and the author notes. The byline should read:

Jeff Pettit, PhD∗, Kristi J. Ferguson, PhD

The author notes should read:

**Jeff Pettit, PhD:** Associate Professor, Department of Family Medicine, University of Iowa Roy J. and Lucille A. Carver College of Medicine

**Kristi J. Ferguson, PhD:** Professor and Director, Office of Consultation and Research in Medical Education, University of Iowa Roy J. and Lucille A. Carver College of Medicine
