# Supplementary material for: Medical Emergency During Flight: A Team-Building Exercise
Source: MedEdPORTAL. 2017 Jan 13;13:10530. doi: 10.15766/mep_2374-8265.10530 (PMC6342154; doi:10.15766/mep_2374-8265.10530)
Supplement: Supplementary file 1 — A. Facilitator's Guide.docx B. Handout.docx C. Evaluation Form.docx [file mep-13-10530-s001.zip › _Educational Summary Report_10530.pdf]

MedEdPORTAL®
The Journal of Teaching and Learning Resources

Crossref

Original Publication

OPEN ACCESS

Medical Emergency During Flight: A Team-Building Exercise

Jeff Pettit, MD*, Kristi J. Ferguson, MD
*Corresponding author: jeffrey-pettit@uiowa.edu

Citation: Pettit J, Ferguson KJ. Medical emergency during flight: a team-building exercise. *MedEdPORTAL*. 2017;13:10530.
https://doi.org/10.15766/mep_2374-8265.10530

Copyright: © 2017 Pettit and Ferguson. This is an open-access article distributed under the terms of the Creative Commons Attribution license.

Appendices

A. Facilitator's Guide.docx
B. Handout.docx
C. Evaluation Form.docx

All appendices are peer reviewed as integral parts of the Original Publication.

Abstract

Introduction: While teams are a central component in health care, many professionals who function in them have had little, if any, formal training on how to develop an effective team. Medical educators and trainers have used many different approaches to teach the basic skills and knowledge of team effectiveness and how team members can best interact with each other. To make team training more realistic, experiential exercises have been used. One of the more popular categories of experiential activities is survival exercises in which team members are given a scenario and required to make decisions that ultimately decide whether the team survives the ordeal. **Methods:** This activity describes a situation in which a medical professional is traveling on an airliner when a request for medical assistance occurs. Participants can include clinically experienced medical students, residents, fellows, and faculty physicians. The activity can be used as a stand-alone exercise or in conjunction with another team topic, such as communications or decision making. It has also been effective as an icebreaker for teams working together during a workshop. **Results:** Approximately 100 medical students, residents, and faculty from anesthesia, family medicine, pediatrics, and internal medicine have participated in this activity. It has been very well received and generated a great deal of discussion of both medical knowledge and team-building skills. **Discussion:** This activity, which can be used to examine team communications, decision making, leadership, and conflict management, is suitable for health care professionals either through intra- or interprofessional training.

Keywords

Leadership, Decision Making, Conflict, Team Building, Communications

Educational Objectives

By the end of this module, the learner will be able to:

1. Identify ways to improve team decision making.
2. Examine how ineffective communication hinders team decision making.
3. Explore how strong leadership or the absence of leadership impacts team decision making.
4. Investigate team decision making in a challenging, interactive, and fun way.
5. Explain why the team's score is almost always better than an individual's score.

Introduction

Teams are an integral component in health care. For many years, different types of teams have been a part of patient care, health care administration, quality improvement, and patient safety. While teams are a central component of health care, many of the professionals who function in these teams have had little, if any, formal training on how to develop an effective team. Medical educators and trainers have used many different approaches to teach the basic skills and knowledge of team effectiveness and how team members can best interact with each other.

To make team training more realistic, experiential exercises have been used. One of the more popular categories of experiential activities is survival exercises. In these types of simulation, team members are given a scenario and then required to make decisions that ultimately decide whether the team survives the ordeal. For example, Yetton and Bottger[1] created the moon survival exercise to investigate group

problem solving. Team members are told that they have crash-landed on the moon far from the home base. They have been able to salvage items from the wreckage that might be used in aiding their survival. The challenge is that first individually and then collectively, the team must rank order the items from most to least important. Their answers are compared to an expert's opinion, which allows them to quantitatively score their results. This type of exercise can be used to improve components of team building such as communications, decision making, leadership, and conflict management.

Survival exercises have been used to investigate many aspects of team effectiveness. Rogelberg and Rumery[2] used the winter survival exercise created by Johnson and Johnson[3] to examine the effects of gender diversity on team decision quality, time on task, and interpersonal cohesion. Jordan and Troth[4] used the subarctic situation, developed by Lafferty and Eady,[5] to assess the impact of emotional intelligence on team performance and conflict resolution methods. We have used the moon crash and lost at sea survival exercises numerous times with physicians in multiple specialties as an icebreaker or to reinforce one of the components of team building. Unfortunately, there were no survival-type exercises that were medically focused. Therefore, one of us (Jeff Pettit) created this activity to be used to support team training in medical environments.

This educational summary report describes a variation on survival activities that is more medically focused. The activity—a medical emergency during flight—depicts a situation in which a medical professional is traveling on an airliner when a request for medical assistance occurs. After responding, the physician is given a first-aid kit with 15 items. There are two challenges to this activity: developing a list of differentials and rank ordering the 15 items to aid in assisting the flight passenger. The rank ordering is done both individually and collectively and then compared to a ranking by experts. The expert rankings were obtained by consensus from three emergency medicine physicians licensed in advanced cardiac life and trauma support and having 30 years' collective experience in emergency medicine.

As with other survival exercises, the team-building components of decision making, leadership, communications, and conflict management can be investigated in this activity through discussion with all participants.

- Decision making: asking each team what method of decision making was used, how this particular method was picked, and how well it worked.
- Leadership: asking each team if someone assumed the role of leader and, if so, how this manifested.
- Communications: asking each team if everyone had equal say and contributed equally, whether any team member did not talk or contribute, and the reasons why.
- Conflict management: asking each team if differences of opinion were resolved amicably, if conflict slowed down the decision making or communications, and if it impacted any team member's involvement.

Insights from these and other responses from participants will provide a better understanding of team dynamics and its impact on team performance. This activity has been used with residents, fellows, and faculty in different medical specialties as part of team training at the University of Iowa Hospitals & Clinics, Iowa City, IA, and UnityPoint Hospital, Des Moines, IA.

Methods

Participants in this activity can include clinically experienced medical students, residents, fellows, and faculty physicians. The exercise has been especially effective with interprofessional groups that included physicians, nurses, quality and safety staff, and pharmacists.

The activity can be used as a stand-alone exercise or in conjunction with another team topic, such as communications or decision making. It has also been effective as an icebreaker for groups that will be working together in teams during a workshop. It takes approximately 40 minutes to complete. As a stand-

alone, the focus would be on the following team dynamics: leadership, conflict management, decision making, communications, or team member interactions. In a support role, the topic could be, for example, communications (different styles), and the exercise would be used to examine different styles within a group or team.

To complete this as a stand-alone module, the facilitator should prep him- or herself to be familiar with the activity and have a copy of the Facilitator's Guide (Appendix A) for debriefing. Next, divide the participants into teams of four to six people. Provide each participant with the handout on the medical emergency during flight (Appendix B). Explain to the participants the background for the activity as outlined in the handout. Give the participants approximately 5 minutes to individually rank order the items in the first column (the facilitator needs to be able to tell when a majority of the participants have completed this step). Give the teams approximately 20 minutes to rank order the items in the second column (the facilitator needs to be able to tell when all of the teams have completed this step). Verbally provide the expert rankings (see Item 3-I in the Facilitator's Guide) to be recorded on the handout in the third column.

Participants can find their individual score in the fourth column. To calculate this score, subtract their individual values from the expert rankings, and calculate the absolute value. Then, add up the list to calculate a total individual score. Participants can find their group score in the fifth column. To calculate this score, subtract the group/team values from the expert rankings, and calculate the absolute value. Then, add up the list to calculate a total group/team score.

Finally, use the Facilitator's Guide to debrief the activity (approximately 10-15 minutes). The learning objectives should be assessed during the debriefing session. Teams should discuss their approach to decision making, team communication, and leadership roles. Finally, teams should complete the postactivity evaluation (Appendix C) to determine whether participants thought the activity was effective when used as an icebreaker or in conjunction with some component of team training.

This activity was first tested on a group of community teaching scholars in support of team training. The activity generated a great deal of discussion related to the medical aspects (differential and use of certain medications) as well as to how each group functioned. Since the first test, it has been used with anesthesia residents, internal medicine faculty and residents, family medicine residents, pediatric residents and rotating students, physicians enrolled in the master's degree in medical education program, and interprofessional groups at simulation debriefing workshops.

A facilitator with no medical training or background can be effective with this exercise. The participants are happy to share their knowledge regarding the differentials and items on the list. Expect there to be a many differences of opinion as to the importance of some items. While this was intended in the development, many specialties have differing perspectives on the use of medications given a certain diagnosis. The participants will likely want to challenge the facilitator on the rankings. The rankings were derived from discussions with three emergency medicine physicians and information on first-aid kits available on airplanes. The debriefing can take more time depending on the discussions around any of the items and expert rankings.

Results

Approximately 100 medical students, residents, and faculty from anesthesia, family medicine, pediatrics, and internal medicine have participated in this activity. It has been very well received and has generated a great deal of discussion of both medical knowledge and team-building skills. The exercise has been used to support team-development topics such as communication and decision making. Evaluations collected from 30 participants in the interprofessional debriefing workshops rated the activity with a mean of 4.56 out of 5 possible points (in response to the question "Please evaluate each presentation on its utility in preparing you to run and debrief interprofessional simulations," with 1 = *poor,* 5 = *outstanding*).

In response to a brief survey sent to participants after the activity, 50% said “strongly agree” and 41% said “agree somewhat” when asked whether the exercise helped them learn about team dynamics; 73% said “strongly agree” and 23% said “agree somewhat” when asked whether it was a fun activity.

Representative comments included the following:

- “We did your exercise this morning at our Wellness Morning Report for the Pediatric Residents and rotating students. It was very well received (although they argued some of what the ‘experts’ said, of course!). The feedback was it was a fun team-building activity, and that they’d like you to develop something pediatric specific!”
- “Was a worthwhile exercise.”
- “It was interesting that since we didn’t really know each other, it was easy to state and share my opinions.”
- “It was a fun way to think about how decisions are made within teams and how individuals with different capabilities and levels of exercise work together to solve problems. The exercise had great practical relevance to show the types of challenges a multidisciplinary interprofessional team encounters in caring for patients.”
- “A great exercise for team building that has a medical application.”
- “I had questions about how the ‘right’ or ‘expert’ answers were chosen, but overall enjoyed the activity, and it helped us practice consensus-building.”
- “I enjoyed participating in the team, collaborating as well as watching leaders emerge.”
- “The exercise was fun. I was impressed how much better the scores were after working in a team.”

Discussion

Medical educators and trainers are often looking for content-appropriate materials to use in various training sessions. One area that has a great deal of material, team development, uses experiential activities in the form of survival exercises to expose and reinforce the topics of communication, decision making, leadership, and conflict management. Unfortunately, there were no survival-type exercises that were medically related. To remedy this, the medical emergency during flight has been specifically designed for medical professionals in conjunction with team training. While the activity works well with most medical specialties, anesthesiologists and pharmacists may have slightly different views on the ranking of the items. The focus should be on the approaches that teams use in determining the ranking. Trainers who use this activity will appreciate that it focuses on both medical knowledge and team dynamics. For the facilitator, there is very little preparation required; the major role for the facilitator is to observe team dynamics and debrief the results. This activity can be used in a stand-alone mode or to support other team topics, such as communication or decision making. Based on the success so far of this activity, we plan to develop other such exercises with a medical focus.

Jeff Pettit, MD: Associate Professor, Department of Family Medicine, University of Iowa Roy J. and Lucille A. Carver College of Medicine

Kristi J. Ferguson, MD: Professor and Director, Office of Consultation and Research in Medical Education, University of Iowa Roy J. and Lucille A. Carver College of Medicine

---

Disclosures
None to report.

Funding/Support
None to report.

Ethical Approval
Reported as not applicable.

References

1. Yetton PW, Bottger PC. Individual versus group problem solving: an empirical test of a best-member strategy. *Organ Behav Hum Perform.* 1982;29(3):307-321. https://doi.org/10.1016/0030-5073(82)90248-3
2. Rogelberg SG, Rumery SM. Gender diversity, team decision quality, time on task, and interpersonal cohesion. *Small Group Res.* 1996;27(1):79-90. https://doi.org/10.1177/1046496496271004
3. Johnson DW, Johnson FP. *Joining Together: Group Theory and Group Skills*. 4th ed. Englewood Cliffs, NJ: Prentice Hall; 1991.
4. Jordan PJ, Troth AC. Managing emotions during team problem solving: emotional intelligence and conflict resolution. *Hum Perform.* 2004;17(2):195-218. https://doi.org/10.1207/s15327043hup1702_4
5. Eady PM, Lafferty JC. *The Subarctic Survival Situation*. Plymouth, MI: Human Synergistics; 1975.

Received: August 12, 2016 | **Accepted:** December 21, 2016 | **Published:** January 13, 2017
