# Supplementary material for: Medical Emergency During Flight: A Team-Building Exercise
Source: MedEdPORTAL. 2017 Jan 13;13:10530. doi: 10.15766/mep_2374-8265.10530 (PMC6342154; doi:10.15766/mep_2374-8265.10530)
Supplement: Supplementary file 1 — A. Facilitator's Guide.docx B. Handout.docx C. Evaluation Form.docx [file mep-13-10530-s001.zip › A. Facilitator's Guide.docx]

Facilitator’s Guide

**1. Items available for this activity**

- Educational Summary Report
- Medical Emergency during Flight handout
- Facilitator’s Guide

**2. Explanation of usage & audience**

This activity can be used as an energizer activity, an icebreaker, or can be used to support team development in the areas of communication, decision making, leadership, or conflict management. The exercise will take approximately 40 minutes, depending on the debriefing. This activity is suitable for clinically experienced medical students, residents, fellows, and faculty physicians in multiple specialties except Anesthesia. Anesthetists have a different approach to the application of some of the drugs identified in this exercise.

3. Methods

1. Prior to the session, make a copy of the Medical Emergency during Flight handout (front and back of one sheet of paper)
2. Explain to the participants that the focus of the session will be multiple aspects of team dynamics and briefly cover the aspects (focus on decision making, communications, conflict management, or leadership)
3. Divide the participants into groups/teams of 4-6 people
4. Provide each participant with the *Medical Emergency during Flight* handout
   1. One side contains the instructions/background for the activity
   2. One side contains five columns for ranking the items
5. Explain to the participants the background for the activity as outlined in the handout. Also explain that you will be giving them expert rankings to compare both team and individual rankings.
6. Give the participants approximately 5 minutes to individually rank order the items in the first column (facilitator will be able to tell when a majority of the participants have completed this step)
7. Give the groups/teams approximately 20 minutes to rank order the items in the second column (facilitator will be able to tell when all of the groups/teams have completed this step)
8. NOTE: As the participants work on this exercise, move around the room to observe any of the following:
   1. How the team is communicating? Are they listening to each other? Talking over each other?
   2. Is everyone participating? Is anyone being excluded?
   3. Did one person take over the role of leader? Was there shared leadership?
   4. Was there any conflict? Was there open disagreement or hidden disgust?
   5. Did the team seem to function well or not very well?
9. Verbally provide the expert rankings (*Facilitator’s Guide*) to be recorded on the handout in the third column

EXPERT EXPLANATION OF RANKINGS

| **ITEM** | **RANKING** | **REASONING** |
| --- | --- | --- |
| Epinephrine, injectable | 12 | Would be more helpful for anaphylaxis, but this is unlikely given the history presented in the scenario, if the patient does cardiac arrest one round of epinephrine might help achieve return of circulation, but this would likely only be a brief temporizing measure (on top of which epinephrine for cardiac arrest does not appear to increase the odds of a patient leaving the hospital either alive or neurologically intact). |
| Benzodiazepine, injectable | 10 | Would be more useful if patient was seizing, but could be used to treat a severe panic attack. |
| Sphygmomanometer | 4 | Determining whether the patient is hypotensive, normotensive or hypertensive provides valuable information about their clinical status (i.e. hypertension with crackles on auscultation = acute pulmonary edema, hypotension suggests shock due to Myocardial Infarction or tamponade). Could inform decision about whether to make an emergency landing. |
| 16 gauge needle with IV catheter & syringe | 3 | Can treat Pneumothorax with needle decompression, this could potentially be lifesaving. Also allows IV access for other meds to be given. In extremely unlikely event of pericardial tamponade could be used for emergent pericardiocentesis. |
| Antiseptic wipes | 7 | Necessary if you’re going to treat a Pneumothorax or insert an IV. |
| Stethoscope | 2 | Helps rule or rule out critical diagnoses including pneumothorax (Pneumothorax) or acute pulmonary edema. |
| One roll of adhesive tape | 8 | Hold in place IV or needle thoracostomy for Pneumothorax. |
| Thermometer | 13 | Temperature is not going to be helpful; if infectious there is little we can do about it at this point. |
| Umbilical cord clamp | 15 | Almost zero use |
| Surgical mask | 14 | Essentially useless, if this patient has an acute infectious process that is contagious, everyone on the plane is likely to already be affected. |
| Endotracheal tube with laryngoscope | 9 | In the absence of paralytics or sedatives would be extremely difficult to intubate patient until they had almost coded, also since there is no mention of a bag to assist respirations, even if patient is intubated it would be nearly impossible to assist his respiration or ventilation, but you could help protect the airway and prevent aspiration in a patient who loses consciousness, could provide manual (lip to tube) respiratory assistance. |
| Aspirin | 1 | Treats Acute Coronary Syndrome with mortality benefit (NNT = 42 for patients with STEMI). |
| Gloves, nitrile | 6 | Necessary if you’re going to have to do any procedures, also finger of glove can be used to create stopcock release valve in case of Pneumothorax. |
| Advanced life support cards | 11 | Can help guide practitioners through code or peri-code scenarios especially if they are unfamiliar or out of practice. |
| Nitroglycerin pills | 5 | If acute pulmonary edema, this could be lifesaving, if Acute Coronary Syndrome will improve symptoms though has no impact on mortality or other outcomes. |

The differential diagnosis, based on the description provided is as follows:

1. Acute Coronary Syndrome (covers everything from Angina to ST Elevation MI)
2. Pneumothorax
3. Pulmonary Embolism
4. Thoracic Aortic Dissection
5. Acute Pulmonary Edema
6. COPD/Asthma Exacerbation
7. Anxiety Attack
8. Allergic Reaction
9. Pericarditis with or without tamponade

This list informs the value of the items in this kit to this particular patient. For example, if the presentation was more concerning for anaphylaxis/allergic reaction, epinephrine would be much higher on the list. This list is based on the ability of the items on the list to provide benefit or treatment for the potential diagnoses in this patient’s differential.

1. Instruct participants to find their individual score in the fourth column:
   1. Subtract their individual values from the expert’s rankings and calculate the absolute value (only positive scoring)
   2. Add up the list to calculate a total individual score
2. Instruct participants to find their group/team score in the fifth column:
   1. Subtract the group/team values from the expert’s rankings and calculate the absolute value (only positive scoring)
   2. Add up the list to calculate a total group/team score
3. Debriefing
   1. Expect some dissension from participants for the rankings (there is usually some push-back). From the facilitator’s point of view, how did the participants deal with the “expert’s” rankings?
   2. Ask all participants for a collective general assessment of the team and individual performance. An interesting place to start is to ask how they approached the task in terms of ranking the items as a team, how they resolved any differences, and whether a leader emerged in this process.
      1. Teams typically start by trying to rank the number 1 item (highest priority) and then the next lowest priority
      2. Teams also start by trying to rank the number 15 item (lowest priority) and then the next highest priority
      3. Teams have been known to place the items into 3 categories—highest priority, medium priority, lowest priority—and then rank within category
   3. Check with each team to see what the team score is (you could reward the team with the lowest score)
      1. Facilitator should ask how each team made its decisions (voting, consensus, dominant person, etc.)
   4. Check with the entire group to see who had the lowest score (you could reward the individual with the lowest score)
   5. Check with the entire group to see who had the highest score (you could reward the individual(s) with the highest score)
   6. Check with each team to see if any individual had a score lower than the team he/she was in
      1. If this does occur, ask the team if they have an explanation for this discrepancy
      2. Likely discrepancies
         1. Person did not speak up
         2. Person tried to talk but others did not listen
         3. Person was not confident in their assessment, so preferred not to speak up
         4. Sometimes there is bias towards women, minorities, etc. to not take them seriously
   7. Ask each team about its assessment of the communication skills within the team and why they think this way
   8. Ask each team about its assessment of the conflict management skills within the team and why they think this way
   9. Ask each team about it assessment of the leadership skills within the team and why they think this way
   10. Ask each team how they made their decisions and why they took this approach
   11. Ask the collective group if there are techniques/methods they could use to improve any of items g – j
   12. Ask if there are any other observations from participants
4. To evaluate the session, use the evaluation form
   1. Get a commitment from everyone to try one of the identified techniques/methods for improvement
