# Supplementary material for: Medical Emergency During Flight: A Team-Building Exercise
Source: MedEdPORTAL. 2017 Jan 13;13:10530. doi: 10.15766/mep_2374-8265.10530 (PMC6342154; doi:10.15766/mep_2374-8265.10530)
Supplement: Supplementary file 1 — A. Facilitator's Guide.docx B. Handout.docx C. Evaluation Form.docx [file mep-13-10530-s001.zip › B. Handout.docx]

Medical Emergency during Flight

You are flying cross-country, on your way to a much needed vacation. An announcement from the flight attendant asks if there is a doctor on board the plane. Being a good Samaritan, you signal that you are medically trained. The flight attendant says that a passenger in first class is complaining of a sudden onset of chest pain. On your way to see the passenger, the flight attendant hands you the on-board emergency medical kit. The passenger is grasping his chest and says it hurts. When you open the kit, there is a checklist of all the items contained in the kit. However, it is obvious that the kit has been used before and missing a great many items. Only the following items are in the kit:

- Epinephrine, injectable
- Benzodiazepine, injectable
- Sphygmomanometer
- 16 gauge needle with IV catheter & syringe
- Antiseptic wipes
- Stethoscope
- One roll of adhesive tape
- Thermometer
- Umbilical cord clamp
- Surgical mask
- Endotracheal tube with laryngoscope
- Aspirin
- Gloves, nitrile
- Advanced life support cards
- Nitroglycerin pills

**Your task:**

1. Individually rank the items in order of importance from most important (1) to least important (15) to help with the patient.
2. As a group, rank the items in order of importance from most important (1) to least important (15) to help with the patient. Also as a group, indicate your top 3 differential diagnoses.

#1 _____________________________________

#2 _____________________________________

#3 _____________________________________

1. Compare your individual and group rankings to those of emergency medicine experts.

Ranking Table

| **ITEM** | **INDIVIDUAL** | **GROUP** | **EXPERT** | **INDIVIDUAL - EXPERT** | **GROUP - EXPERT** |
| --- | --- | --- | --- | --- | --- |
| Epinephrine, injectable |  |  |  |  |  |
| Benzodiazepine, injectable |  |  |  |  |  |
| Sphygmomanometer |  |  |  |  |  |
| 16 gauge needle with IV catheter & syringe |  |  |  |  |  |
| Antiseptic wipes |  |  |  |  |  |
| Stethoscope |  |  |  |  |  |
| One roll of adhesive tape |  |  |  |  |  |
| Thermometer |  |  |  |  |  |
| Umbilical cord clamp |  |  |  |  |  |
| Surgical mask |  |  |  |  |  |
| Endotracheal tube with laryngoscope |  |  |  |  |  |
| Aspirin |  |  |  |  |  |
| Gloves, nitrile |  |  |  |  |  |
| Advanced life support cards |  |  |  |  |  |
| Nitroglycerin pills |  |  |  |  |  |
|  | | | **TOTAL** |  |  |
