# Supplementary material for: Medical Emergency During Flight: A Team-Building Exercise
Source: MedEdPORTAL. 2017 Jan 13;13:10530. doi: 10.15766/mep_2374-8265.10530 (PMC6342154; doi:10.15766/mep_2374-8265.10530)
Supplement: Supplementary file 1 — A. Facilitator's Guide.docx B. Handout.docx C. Evaluation Form.docx [file mep-13-10530-s001.zip › C. Evaluation Form.docx]

**Medical Emergency during Flight Evaluation Form**

|  | Strongly Disagree | |  | Strongly  Agree | |
| --- | --- | --- | --- | --- | --- |
| 1. This exercise helped me learn about team dynamics. | 1 | 2 | 3 | 4 | 5 |
| 2. This was a fun activity. | 1 | 2 | 3 | 4 | 5 |

3. Identify 1-2 concepts that you learned from this session that will help you and your team(s).

4. Specifically, how could you apply this new concept(s) in your role as a team leader?

5. Was there 1 concept from this session that needs more explanation and/or in-depth discussion?

6. General comments/feedback:
